# Tremor, Daily Functioning, and Health-Related Quality of Life in Solid Organ Transplant Recipients

**DOI:** 10.3389/ti.2023.10951

**Published:** 2023-03-16

**Authors:** Niels L. Riemersma, Daan Kremer, Tim J. Knobbe, C. Tji Gan, Svea Nolte, António W. Gomes-Neto, Hans Blokzijl, Vincent E. de Meijer, Kevin Damman, Michele F. Eisenga, Gea Drost, Jan Willem J. Elting, Daan J. Touw, Stefan P. Berger, Stephan J. L. Bakker, A. M. Madelein van der Stouwe, Transplantlines Investigators, Coby Annema

**Affiliations:** ^1^ Division of Nephrology, Department of Internal Medicine, University Medical Center Groningen, University of Groningen, Groningen, Netherlands; ^2^ Department of Pulmonary Diseases and Lung Transplantation, University Medical Center Groningen, University of Groningen, Groningen, Netherlands; ^3^ Department of Gastroenterology and Hepatology, University Medical Center Groningen, University of Groningen, Groningen, Netherlands; ^4^ Department of Hepato-Pancreato-Biliary Surgery and Liver Transplantation, University Medical Center Groningen, University of Groningen, Groningen, Netherlands; ^5^ Department of Cardiology, University Medical Center Groningen, University of Groningen, Groningen, Netherlands; ^6^ Department of Neurology and Clinical Neurophysiology, University Medical Center Groningen, University of Groningen, Groningen, Netherlands; ^7^ Department of Pharmaceutical Analysis, Groningen Research Institute of Pharmacy, University of Groningen, Groningen, Netherlands; ^8^ Department of Clinical Pharmacy and Pharmacology, University Medical Center Groningen, University of Groningen, Groningen, Netherlands; ^9^ Expertise Center Movement Disorders Groningen, University Medical Center Groningen, University of Groningen, Groningen, Netherlands

**Keywords:** tremor, solid organ transplant recipient, calcineurin inhibitors, health-related quality of life, ADL impairment

## Abstract

Solid organ transplant recipients (SOTR) frequently report tremor. Data concerning tremor-related impairment and its potential impact on health-related quality of life (HRQoL) are lacking. This cross-sectional study assesses impact of tremor on activities of daily living and HRQoL using validated questionnaires among SOTR enrolled in the TransplantLines Biobank and Cohort Study. We included 689 SOTR (38.5% female, mean [±SD] age 58 [±14] years) at median [interquartile range] 3 [1–9] years after transplantation, of which 287 (41.7%) reported mild or severe tremor. In multinomial logistic regression analyses, whole blood tacrolimus trough concentration was an independent determinant of mild tremor (OR per µg/L increase: 1.11, 95% CI: 1.02 to 1.21, *p* = 0.019). Furthermore, in linear regression analyses, severe tremor was strongly and independently associated with lower physical and mental HRQoL (β = −16.10, 95% CI: −22.23 to −9.98, *p* < 0.001 and β = −12.68, 95% CI: −18.23 to −7.14, *p* < 0.001 resp.). SOTR frequently report tremor-related impairment of activities of daily living. Tacrolimus trough concentrations appeared as a main determinant of tremor among SOTR. The strong and independent association of tremor-related impairment with lower HRQoL warrants further studies into the effects of tacrolimus on tremor.

**Clinical Trial Registration**: ClinicalTrials.gov, Identifier NCT03272841.

## Graphical Abstract

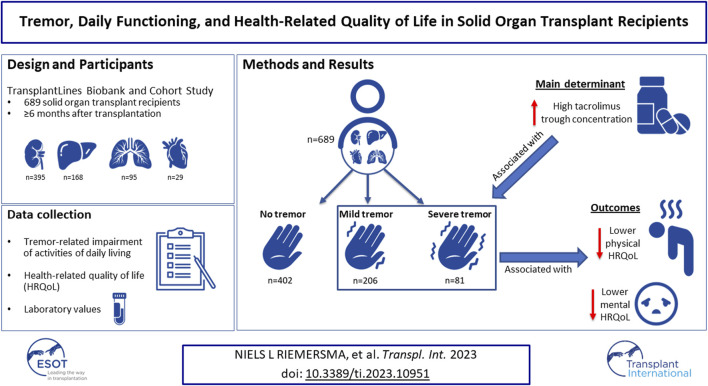

## Introduction

Solid organ transplantation has evolved from a scientific novelty to the preferred treatment for end-stage organ failure. For example, kidney, liver, lung, and heart transplantations strongly improve long-term survival in otherwise untreatable diseases (1–7). Unfortunately, even after successful transplantation, solid organ transplant recipients (SOTR) continue to have reduced health-related quality of life (HRQoL) compared to the general population (8–10). A suggested cause for this limited HRQoL is the need to adhere to a strict immunosuppressive maintenance regime, generally including calcineurin inhibitors (CNIs) (11). These CNIs, including cyclosporine and tacrolimus, are essential to prevent graft rejection, and are a cornerstone in current post-transplantation care (12). However, CNI-use is associated with multiple side effects, including nephrotoxicity and neurotoxicity (13, 14).

One of the most frequently reported side effects of CNIs is the development of tremor: rhythmic, sinusoidal oscillations of the limbs, head, or trunk (15). Medication-induced tremor may limit SOTR in performing activities of daily living (ADL), such as eating, writing, and personal hygiene, much like tremor interferes with ADL in other populations (16).

CNI-induced tremor generally occurs soon after initiation of CNI maintenance therapy (17), and occurs in up to half of SOTR using CNIs (18). The occurrence of CNI-induced tremor is reportedly dose-dependent, yet some patients experience tremor even at low dosages (18, 19). Currently, we lack the knowledge to what extent tremor leads to impairments in ADL, and how this may affect HRQoL among SOTR. In-depth investigation of the impact of tremor on ADL and HRQoL is therefore warranted and may add valuable information to previous studies that abstained from using tremor-related ADL impairment as primary outcome measurement (18).

We therefore aimed to assess the prevalence and severity of tremor-related ADL impairment among SOTR. Additionally, we aimed to identify clinical, biochemical, and pharmaceutical factors that may predispose SOTR for the development of tremor-related impairment. Finally, we assessed associations of tremor-related impairment with HRQoL.

## Materials and Methods

### Design and Study Population

For this cross-sectional study, data from the TransplantLines Biobank and Cohort Study (ClinicalTrials.gov Identifier: NCT03272841) were used (20). This ongoing cohort study includes SOTR and donors (aged ≥18 years) visiting the University Medical Center Groningen (UMCG, Groningen, The Netherlands). More detailed information on the study design, inclusion and exclusion criteria has been described previously (20). The study was conducted in accordance with the guidelines laid down in the Declarations of Helsinki and Istanbul and approved by the Institutional Review Board (METc 2014/077). The study protocol was reviewed and approved by the TransplantLines Scientific Committee (TxL 2022/004).

For the current study, all enrolled SOTR with a functioning allograft for at least 6 months, with available data on tremor influence on ADL between September 2016 and November 2020, were included. A consort flow diagram is shown in Supplementary Figure S1.

### Immunosuppressive Regimen

All included SOTR attended the outpatient clinic of the UMCG and were treated according to standard immunosuppressive therapies, with revision of therapy effectiveness at least once per year. Immunosuppressive maintenance therapies were generally tacrolimus-based, with addition of mycophenolate mofetil (MMF), and prednisolone. Other immunosuppressive drugs used less frequently were cyclosporine, everolimus, sirolimus, and azathioprine. Although immunosuppressive regimens may be adapted on an individual basis, standard protocol target trough concentrations of tacrolimus and cyclosporine in the UMCG are shown in Table 1.

**TABLE 1:** Immunosuppressive target trough concentration per transplant type in the UMCG.

Time after transplantation (months)	Tacrolimus trough concentration (µg/L)			Cyclosporine trough concentration (µg/L)		
	7–12	13–24	>24	7–12	13–24	>24
Kidney transplantation	4–6	4–6	4–6	75–125	75–125	75–125
Liver transplantation						
^a^Dual therapy	7–10	7–10	7–10	*	*	*
^b^Triple therapy	5–7	5–7	5–7	*	*	*
Lung transplantation	7–10	7–10	7–10	150	100–150	100
Heart transplantation	7–9	7–9	7–9	150	125	125
Small intestine transplantation	10–15	10–15	10–15	∼	∼	∼

^a^Dual therapy consists of tacrolimus and prednisone, and ^b^triple therapy additionally includes mycophenolate mofetil.

∼ target trough concentrations are not listed in UMCG protocols. *Cyclosporine is not used for this type of transplantation.

### Laboratory Methods

Blood was drawn in the morning after an overnight fasting period of at least 8 h, including no medication use. All tacrolimus and cyclosporine whole blood trough concentrations were determined by validated liquid chromatography mass-spectrometry analyses at the UMCG (21). Other laboratory parameters were measured using routine laboratory methods (Roche Diagnostics, Basel, Switzerland).

### Tremor Rating

Tremor severity was assessed using a Dutch translation of the Fahn-Tolosa-Marin (FTM) tremor rating scale part C (TRS-C) (22). This questionnaire is recommended for use in clinical practice (23). The TRS-C consists of eight questions to assess patient-perceived tremor occurrence during ADL, including writing, speaking, and bringing food or liquids to the mouth, and is provided in Supplementary Table S1. Furthermore, the questionnaire assesses the influence of tremor on personal hygiene, dressing, working, and social activities. As the TRS-C is designed to evaluate the impact of tremor on ADL, by nature, this questionnaire assesses severity over the previous couple of days of the patient’s life. The interviewers were trained to conduct the questionnaire during routine follow-up visits, and to interpret the answers of patients. To every question, patients graded the impairment tremor has on ADL with a score ranging from 0 (no influence of tremor on ADL) to 4 (severe influence of tremor on ADL). A total score was calculated by summing the individual scores, with a theoretical maximum score of 32 points. Patients with a TRS-C total score of 0, or with a TRS-C total score of 1 acquired because of mild disability of speech (i.e., shaky voice only when nervous), were classified as having no tremor. For data visualisation, and statistical analyses, tremor severity was rated according to severity: no tremor (defined as 0 points or 1 point on speech (“mild tremor only perceived when nervous” on the total TRS C score)), mild tremor (1–3 points), and severe tremor (≥4 points).

### Health-Related Quality of Life

HRQoL was assessed using the 36-Item Short Form health survey, which is a validated questionnaire to assess several health domains, including physical functioning, role limitations due to physical health, role limitations due to emotional problems, vitality, emotional wellbeing, social functioning, pain, and general health perceptions (24). Subsequently, physical component summary (PCS) and mental component summary (MCS) scores were derived, with a higher score meaning a higher HRQoL. The PCS includes items from physical functioning, role limitations due to physical health, vitality, social functioning, pain, and general health perceptions, whereas the MCS includes items from role limitations due to emotional problems, vitality, emotional wellbeing, social functioning, and general health perceptions.

### Additional Data Collection

Blood pressure and heart rate were measured with a semi-automatic device (Philips Suresign VS2+, Andover, Massachusetts, USA). Body weight and height were measured with participants wearing indoor clothing without shoes. Body mass index (BMI) was calculated as weight in kilograms divided by height in meters squared (kg/m^2^). Information on patients’ medical history was extracted from electronic patient records, and medication use was extracted from patient records and verified with the patient by a trained researcher. Diabetes was defined based on use of antidiabetic drugs, fasting glucose ≥7.0 mmol/L, and/or HbA1c ≥6.5%. Kidney glomerular filtration rate was estimated with the creatinine-based CKD-EPI equation (25). Anaemia was defined as a haemoglobin concentration <8.1 mmol/L for men and <7.5 mmol/L for women according to WHO criteria (26).

### Statistical Analyses

Dispersion of TRS-C scores in total, and per transplant type, were visualized by means of pie charts and bar charts. Patient characteristics are presented and compared in patients without tremor, with mild tremor, and with severe tremor. Continuous variables are summarized as mean ± SD or median [interquartile range], depending on distribution, whereas categorical or dichotomous variables are presented as count (%). To assess differences between tremor severity groups, Analyses of Variance were used for normally distributed variables, Kruskal-Wallis tests for non-normally distributed variables and Chi-squared tests for categorical variables. To assess associations of tremor severity with clinical, biochemical and pharmacotherapeutic parameters, multinomial logistic regression analyses, adjusted for sex, age, and log_2_ time after transplantation were performed. Sensitivity analyses with additional adjustment for tacrolimus trough concentrations were performed. Also, exploratory subgroup analyses were performed to compare non-CNI users with no, mild, or severe tremor.

Differences in PCS and MCS between grades of tremor severity were visualised with boxplots. Analyses of variance were performed for testing significance of differences between the different grades. Furthermore, bivariable linear regression analyses with PCS and MCS as dependent variable were performed to assess associations of mild and severe tremor with HRQoL. In multivariable linear regression analyses we assessed the association of mild and severe tremor with HRQoL, while adjusting for potential confounders including age, sex, type of transplantation, log_2_ time after transplantation, polypharmacy, diabetes, anaemia, eGFR, use of tacrolimus, use of cyclosporine, employment status, educational level, and the presence of a partner. Potential presence of effect modification by age and sex was assessed by adding interaction terms to the linear regression models.

Scatterplots and QQ-plots were visually evaluated to assess data distribution. Non-normally distributed variables were transformed using a binary logarithm (log_2_) when necessary to meet the assumptions for regression. All statistical analyses were performed with IBM SPSS software (version 23.0, SPSS Inc., Chicago, IL, USA) and R (version 3.5.1, Vienna, Austria). In all analyses, a two-sided *p*-value <0.05 was considered statistically significant.

## Results

### Baseline Characteristics

In total, 689 SOTR were included in the current study, including kidney (*n* = 395, 57.3%), liver (*n* = 168, 24.4%), lung (*n* = 95, 13.8%), heart (*n* = 29, 4.2%), and small intestine (*n* = 2, 0.3%) transplant recipients with a mean ± SD age of 58.0 ± 13.7 years, of whom 38.5% were female. A detailed overview of baseline characteristics in patients with either no, mild, or severe tremor, is presented in Table 2. In brief, patients with more tremor-related impairment were more often lung transplant recipients, less often liver transplant recipients, more frequently used tacrolimus and tended to have higher tacrolimus trough concentrations. Furthermore, tremor-related impairment was more frequently reported in patients with polypharmacy, patients with lower eGFR, and patients with higher fasting glucose concentrations. Notably, patients with mild or severe tremor tended to have higher cyclosporin trough concentrations, although differences between the groups were not statistically significant.

**TABLE 2:** Characteristics of 689 SOTR without tremor, with mild tremor, or with severe tremor, based on the TRS-C total score.

*Variables*	No tremor (*n* = 402)	Mild tremor (*n* = 206)	Severe tremor (*n* = 81)	*p*-value
Tremor rating scale-C score	0 [0-0]	2.0 [1.0–2.0]	5.0 [4.0–7.0]	-
Recipient				
Female sex	158 (39.3%)	75 (36.4%)	32 (39.5%)	0.7
Age at visit (years)	54.4 ± 13.6	54.9 ± 14.2	58.1 ± 13.0	0.1
Transplant type*				
Kidney (n = 395)	238 (59.2%)	112 (54.4%)	45 (55.6%)	0.5
Liver (n = 168)	**111 (27.6%)**	**44 (21.4%)**	**13 (16.0%)**	**0.042**
Lung (n = 95)	**40 (10.0%)**	**40 (19.4%)**	**15 (18.5%)**	**0.002**
Heart (n = 29)	13 (3.2%)	9 (4.4%)	7 (8.6%)	0.1
Small intestine (n = 2)	0 (100.0%)	1 (0.5%)	1 (1.2%)	0.1
Polypharmacy (>4 drugs)	**340 (84.6%)**	**183 (88.8%)**	**77 (95.1%)**	**0.025**
Diabetes	101 (25.1%)	54 (26.2%)	21 (25.9%)	1.0
Anaemia	81 (24.7%)	52 (30.4%)	25 (35.7%)	0.1
Body mass index (kg/m^2^)	27.0 ± 4.8	26.6 ± 4.7	27.0 ± 5.3	0.5
Kidney transplant characteristics				
Donor age (years)	51.0 [39.0–59.0]	52.0 [40.0–62.0]	51.0 [34.8–61.0]	0.2
Living donor	134 (33.3%)	71 (34.5%)	24 (29.6%)	0.7
Time after transplantation (years)	3.0 [1.0–10.0]	2.0 [0.0–9.0]	2.0 [1.0–11.0]	0.1
Laboratory measurements				
eGFR creatinine (mL/min/1.73m^2^)	**59.6 ± 22.4**	**55.9 ± 20.3**	**53.8 ± 24.2**	**0.041**
Creatinine (µmol/L)	112.0 [89.3–139.8]	118.0 [98.0–140.0]	116.0 [88.0–168.0]	0.1
Haemoglobin (mmol/L)	8.3 ± 1.1	8.1 ± 1.2	8.1 ± 1.2	0.1
HbA1c (mmol/mol)	38.0 [34.0–43.0]	39.0 [35.0–44.8]	39.0 [35.0–44.3]	0.2
Glucose (mmol/L)	**5.6 [5.1–6.5]**	**5.8 [5.2–6.8]**	**5.8 [5.3–7.0]**	**0.030**
Vitamin B12 (pmol/L)	305.0 [245.0–418.5]	307.5 [239.5–422.0]	313.0 [223.0–467.5]	0.9
Folic acid (nmol/L)	13.8 [9.9–18.1]	12.8 [10.0–17.9]	12.3 [9.6–20.7]	0.4
^a^Tacrolimus (µg/L)	**5.7 ± 2.4**	**6.3 ± 2.5**	**6.3 ± 2.1**	**0.022**
^b^Cyclosporine (µg/L)	65.0 [49.0–98.0]	104.0 [83.5–111.0]	88.0 [36.5–120.3]	0.1
Medication				
*Calcineurin inhibitor use*	341 (84.8%)	183 (88.8%)	70 (86.4%)	0.4
No use	61 (15.2%)	23 (11.2%)	11 (13.6%)	0.4
Tacrolimus	**290 (72.1%)**	**170 (82.5%)**	**64 (79.0%)**	**0.014**
Cyclosporine	48 (11.9%)	13 (6.3%)	7 (8.6%)	0.1
mTOR inhibitor	21 (5.2%)	13 (6.3%)	5 (6.2%)	0.8
Proliferation inhibitor	309 (76.9%)	165 (80.1%)	67 (82.7%)	0.4
Prednisolone or prednisone	322 (80.1%)	172 (83.5%)	72 (88.9%)	0.1
Beta blockers	161 (40.0%)	84 (41.0%)	35 (43.2%)	0.9
Short or long acting bronchodilators	20 (5.0%)	10 (4.9%)	6 (7.4%)	0.6

*Percentages were calculated by dividing the number of patients in each transplant type by the total number of all solid organ transplant patients with no/mild/severe tremor. Bold type indicates significance of results.

eGFR, estimated glomerular filtration rate as calculated using CKD-EPI formula. Normally distributed data are presented as mean ± standard deviation, skewed data as median [interquartile range], and categorical data as number (valid percentage). *p*-values represent significance of differences between tremor severity groups as assessed with Analyses of Variance, Kruskal-Wallis or Chi-squared tests, depending on distribution. Data were available in ^a^514 and ^b^62 patients.

### Occurrence of Tremor and Impact on Activities of Daily Life

**FIGURE 1:**
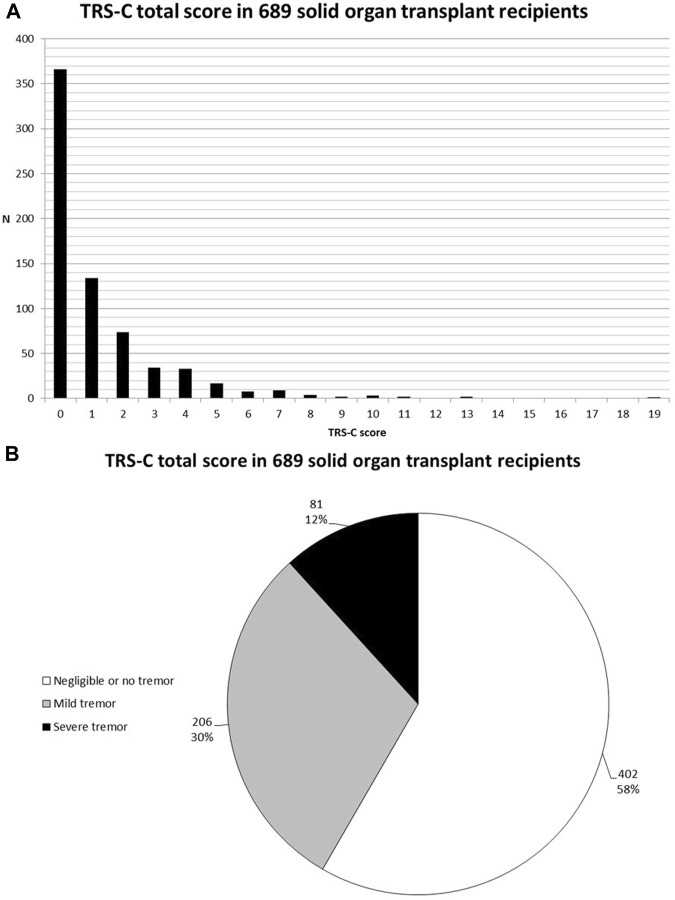
**(A)** Bar chart of TRS-C total score in 689 SOTR. **(B)** Pie-chart of tremor severity in 689 SOTR.

**FIGURE 2:**
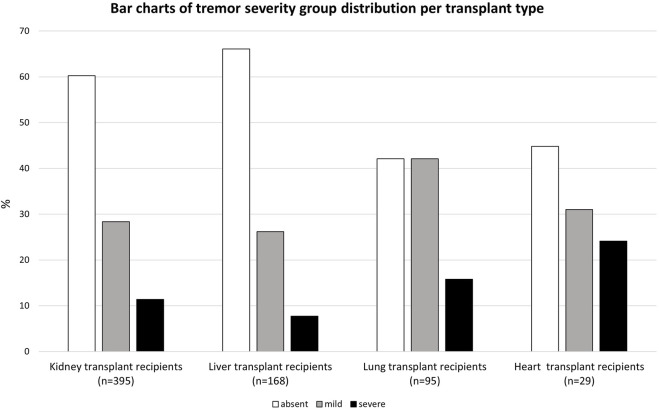
Bar charts of tremor severity group distribution per transplant type.

206 (29.9%) SOTR reported mild tremor and 81 (11.8%) reported severe tremor. Mild or severe tremor was reported in 39.7% among kidney transplant recipients, 33.9% among liver transplant recipients, 57.9% among lung transplant recipients, and 55.1% among heart transplant recipients. TRS-C scores of all SOTR and per organ type are shown in Figures 1A, B and 2 respectively. Additional bar charts of TRS-C total score per organ type are shown in Supplementary Figures S2A–D. Among patients that reported impairment because of tremor, median TRS-C score was 2.0 [1.0–4.0] [range 0–19]. Notably, patients reported that tremor-related impairment was most pronounced during writing and bringing food to the mouth. Furthermore, 18 (2.6%) patients reported changes in social activities because of tremor. Scores per TRS-C question are visualized by means of bar charts in Supplementary Figures S3A–H.

### Determinants of Tremor-Related Impairment

Results of multinomial logistic regression analyses adjusted for age, sex, and log_2_ time after transplantation with presence of mild or severe tremor as dependent variable are shown in Table 3. Whole blood tacrolimus trough concentration was a determinant of mild tremor, independent of age, sex, and log_2_ time after transplantation (OR per μg/L increase: 1.11, 95% CI: 1.02 to 1.21, *p* = 0.019). Notably, higher whole blood tacrolimus trough concentration also tended to be associated with more severe tremor, although this observation was not statistically significant (OR per μg/L increase: 1.11, 95% CI: 0.98 to 1.26, *p* = 0.1). Furthermore, lung transplantation was associated with mild tremor; heart transplantation, polypharmacy and higher creatinine were associated with severe tremor. These associations remained similar after adjustment for tacrolimus trough concentrations (Supplementary Table S2). Notably, of the 95 patients who did not use calcineurin inhibitors, 34 (36%) reported mild or severe tremor. Exploratory subgroup analyses showed that non-CNI users with tremor more often had diabetes (Supplementary Table S3). Use of concomitant medication that may affect tremor, such as beta blockers and bronchodilators, was not associated with mild of severe tremor.

**TABLE 3:** Multinomial logistic regression analyses of tremor severity with adjustment for age, sex, and log_2_ time after transplantation in 689 solid organ transplant recipients.

Baseline variables	No tremor (n = 402)	Mild tremor (*n* = 206)		Severe tremor (*n* = 81)		N
		OR (95% CI)	*p*-value	OR (95% CI)	*p*-value	
Recipient						
*Female sex	*Ref.*	0.91 (0.64–1.29)	0.6	1.05 (0.64–1.71)	0.9	689
*Age at visit (per 10 years)	*Ref.*	1.03 (0.91–1.16)	0.7	**1.23 (1.02–1.48)**	**0.029**	689
Transplant type						
Kidney (*n* = 269)	*Ref.*	*Ref*.	*Ref.*	*Ref.*	*Ref.*	-
Liver (*n* = 154)	*Ref.*	0.98 (0.63–1.53)	0.9	0.64 (0.32–1.27)	0.2	689
Lung (*n* = 75)	*Ref.*	**2.21 (1.34–3.63)**	**0.002**	1.96 (1.00–3.87)	0.1	689
Heart (*n* = 29)	*Ref.*	1.63 (0.67–3.96)	0.3	**2.74 (1.02–7.38)**	**0.047**	689
Polypharmacy (>4 drugs)	*Ref.*	1.26 (0.73–2.15)	0.4	**3.13 (1.08–9.08)**	**0.036**	689
Diabetes	*Ref.*	1.08 (0.73–1.59)	0.7	0.94 (0.54–1.65)	0.8	689
Anaemia	*Ref.*	1.30 (0.86–1.96)	0.2	1.70 (0.98–2.97)	0.1	569
Body mass index (kg/m^2^)	*Ref.*	0.98 (0.94–1.01)	0.2	0.99 (0.94–1.04)	0.8	686
Kidney transplant characteristics						
log_2_ donor age (years)	*Ref.*	1.23 (0.90–1.68)	0.2	0.87 (0.59–1.27)	0.5	642
Living donor	*Ref.*	0.95 (0.66–1.38)	0.8	0.85 (0.50–1.46)	0.6	689
*log_2_ time after transplantation (years)	*Ref.*	**0.90 (0.81–0.99)**	**0.036**	0.96 (0.83–1.11)	0.6	689
Laboratory measurements						
eGFR creatinine (mL/min/1.73m^2^)	*Ref.*	0.99 (0.99–1.00)	0.1	0.99 (0.98–1.00)	0.2	650
log_2_ creatinine (µmol/L)	*Ref.*	1.24 (0.88–1.76)	0.2	**1.71 (1.08–2.70)**	**0.022**	650
Haemoglobin (mmol/L)	*Ref.*	0.87 (0.74–1.02)	0.1	0.82 (0.65–1.03)	0.1	649
log_2_ HbA1c (mmol/mol)	*Ref.*	1.70 (0.97–2.97)	0.1	1.39 (0.62–3.12)	0.4	645
log_2_ glucose (mmol/L)	*Ref.*	1.54 (0.92–2.56)	0.1	1.64 (0.82–3.29)	0.2	624
log_2_ vitamin B12 (pmol/L)	*Ref.*	1.02 (0.78–1.32)	0.9	1.12 (0.78–1.60)	0.6	636
log_2_ folic acid (nmol/L)	*Ref.*	0.97 (0.75–1.25)	0.8	1.08 (0.75–1.55)	0.7	577
Tacrolimus (µg/L)	*Ref.*	**1.11 (1.02–1.21)**	**0.019**	1.11 (0.98–1.26)	0.1	514
log_2_ cyclosporine (µg/L)	*Ref.*	2.12 (0.94–4.81)	0.1	1.32 (0.46–3.75)	0.6	62
Medication						
*Calcineurin inhibitor use*	*Ref.*	1.17 (0.66–2.06)	0.6	1.11 (0.51–2.40)	0.8	689
No use	*Ref.*	*Ref.*	*Ref.*	*Ref.*	*Ref.*	-
Tacrolimus	*Ref.*	1.49 (0.83–2.68)	0.2	1.38 (0.62–3.08)	0.4	689
Cyclosporine	*Ref.*	0.74 (0.34–1.62)	0.5	0.82 (0.29–2.28)	0.7	689
mTOR inhibitor	*Ref.*	1.17 (0.57–2.40)	0.7	1.17 (0.42–3.23)	0.8	689
Proliferation inhibitor	*Ref.*	1.10 (0.72–1.68)	0.7	1.42 (0.75–2.68)	0.3	689
Prednisolone or prednisone	*Ref.*	1.16 (0.74–1.81)	0.5	1.92 (0.91–4.05)	0.1	689
Beta blockers	*Ref.*	0.97 (0.68–1.38)	0.9	1.04 (0.64–1.71)	09	689
Short or long acting bronchodilators	*Ref.*	0.95 (0.43–2.08)	0.9	1.39 (0.54–3.62)	0.50	689

Bold type indicates significance of results. log_2_, the binary logarithm; eGFR, estimated glomerular filtration rate as calculated using CKD-EPI formula. Intestinal transplant recipients are excluded in analyses due to low number of participants (*n* = 2). *Presented numbers represent regression coefficients of the concerned variable in a model including age, sex, and log_2_ time after transplantation.

### Health-Related Quality of Life

**FIGURE 3:**
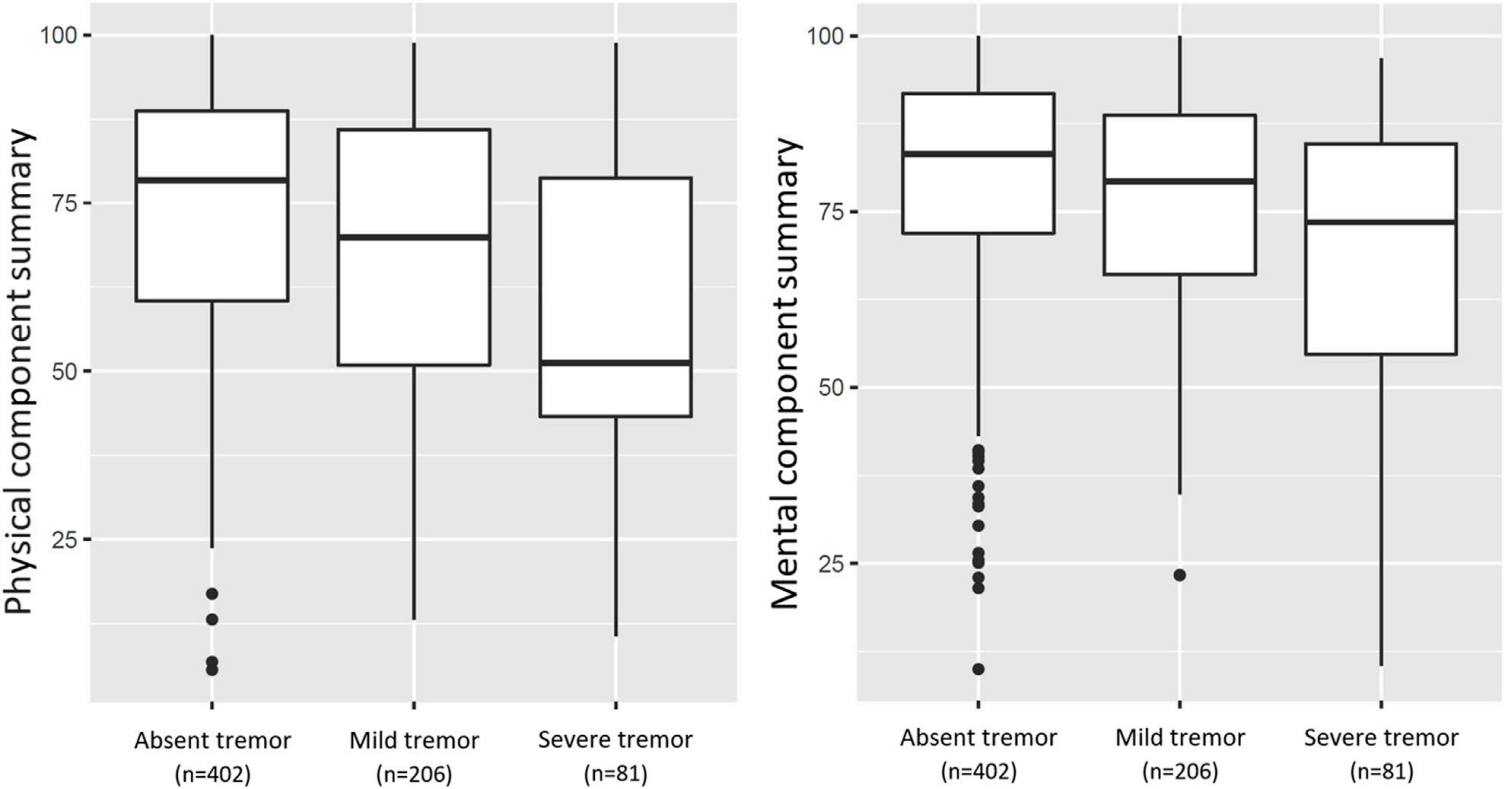
Boxplots of PCS, and MCS for HRQoL in 689 SOTR (mild and severe tremor present in 206 (29.9%) and 81 (11.8%) SOTR, respectively). The PCS, and MCS differed significantly between groups as calculated using Analyses of Variance (*p* < 0.001; *p* < 0.001, resp.).

SOTR that reported mild or severe tremor had significantly lower physical and mental HRQoL, as visualized in Figure 3 (*p* < 0.001 for both). In bivariable linear regression analyses, both mild and severe tremor were associated with a lower physical HRQoL, with the strongest association for severe tremor (PCS: β = −5.64, 95% CI: −9.34 to 1.94, *p* = 0.003 and β = −15.87, 95% CI: −21.26 to −10.48, *p* < 0.001 resp.). A similar pattern was found for mental HRQoL (MCS: β = −3.50, 95% CI: −6.68 to −0.31, *p* = 0.032 and β = −11.22, 95% CI: −15.86 to −6.57, *p* < 0.001 resp.). These associations remained similar after adjustment for age, sex, type of transplantation, log_2_ time after transplantation, polypharmacy, diabetes, anaemia, eGFR, use of tacrolimus, and use of cyclosporine (Table 4). When we additionally adjusted for employment status, educational level, and the presence of a partner, results remained generally similar, although the association between mild tremor and physical HRQoL was no longer statistically significant. No interactions of age and sex were present for the association of tremor severity with HRQoL.

**TABLE 4:** Linear regression analyses of tremor severity with physical and mental HRQoL.

Independent variable	Physical component summary		Mental component summary	
	β (95% CI)	*p*-value	β (95% CI)	*p*-value
Crude				
No tremor	*Ref.*	*n/a*	*Ref.*	*n/a*
Mild tremor	−5.64 (−9.34 to −1.94)	0.003	−3.50 (−6.68 to −0.31)	0.032
Severe tremor	−15.87 (−21.26 to −10.48)	<0.001	−11.22 (−15.86 to −6.57)	<0.001
Model 1				
No tremor	*Ref.*	*n/a*	*Ref.*	*n/a*
Mild tremor	−5.76 (−9.42 to −2.10)	0.002	−3.70 (−6.86 to −0.54)	0.022
Severe tremor	−15.14 (−20.50 to −9.79)	<0.001	−11.54 (−16.16 to −6.93)	<0.001
Model 2				
No tremor	*Ref.*	*n/a*	*Ref.*	*n/a*
Mild tremor	−5.18 (−8.88 to −1.49)	0.006	−3.58 (−6.79 to −0.38)	0.028
Severe tremor	−14.36 (−19.76 to −8.97)	<0.001	−11.41 (−16.09 to −6.73)	<0.001
Model 3				
No tremor	*Ref.*	*n/a*	*Ref.*	*n/a*
Mild tremor	−4.97 (−8.62 to −1.31)	0.008	−3.52 (−6.72 to −0.31)	0.032
Severe tremor	−13.83 (−19.17 to −8.48)	<0.001	−11.24 (−15.93 to −6.56)	<0.001
Model 4				
No tremor	*Ref.*	*n/a*	*Ref.*	*n/a*
Mild tremor	−4.89 (−8.78 to −1.01)	0.014	−3.70 (−7.21 to −0.20)	0.038
Severe tremor	−15.08 (−20.61 to −9.56)	<0.001	−11.89 (−16.87 to −6.90)	<0.001
Model 5				
No tremor	*Ref.*	*n/a*	*Ref.*	*n/a*
Mild tremor	−4.86 (−8.75 to −0.96)	0.015	−3.74 (−7.25 to −0.23)	0.037
Severe tremor	−15.05 (−20.59 to −9.52)	<0.001	−11.92 (−16.91 to −6.92)	<0.001
Model 6				
No tremor	*Ref.*	*n/a*	*Ref.*	*n/a*
Mild tremor	−5.03 (−8.92 to −1.13)	0.012	−3.80 (−7.32 to −0.28)	0.034
Severe tremor	−15.35 (−20.88 to −9.81)	<0.001	−12.06 (−17.06 to −7.06)	<0.001
Model 7				
No tremor	*Ref.*	*n/a*	*Ref.*	*n/a*
Mild tremor	−4.09 (−8.42 to 0.24)	0.06	−4.05 (−7.97 to −0.13)	0.043
Severe tremor	−16.10 (−22.23 to −9.98)	<0.001	−12.68 (−18.23 to −7.14)	<0.001

95% CI, 95% confidence interval; PCS, physical component summary; MCS, mental component summary; eGFR, estimated glomerular filtration rate; HRQoL, health-related quality of life. **Model 1**: adjusted for age and sex; **model 2**: model 1 + type of transplantation and log_2_ time after transplantation; **model 3**: model 2 + polypharmacy; **model 4**: model 3 + diabetes and anaemia; **model 5**: model 4 + eGFR; **model 6**: model 5 + use of tacrolimus and use of cyclosporine; **model 7**: model 6 + employment status, education level, and presence of a partner.

## Discussion

This study shows that mild or severe tremor frequently impairs daily life activities of SOTR. Tacrolimus trough concentration was a main determinant of tremor. Importantly, mild and severe tremor-related impairments were strongly associated with lower HRQoL, independent of other known determinants of tremor.

Our results confirm that SOTR frequently experience tremor-related impairment of ADL. A previous study reported similar tremor prevalence compared to our study (18). However, the current study provides additional insights, because we assessed prevalence based on impact of tremor on ADL. The pathophysiology of CNI-induced tremor remains unknown. However, the transplant population tends to show lower TRS-C scores compared to patients with essential tremor (27). Our results show that tacrolimus trough concentrations are associated with tremor-related impairment among SOTR. This is further corroborated by the finding that tremor-related impairment is higher among SOTR with higher target trough concentrations (e.g., lung and heart transplant recipients). Also, patients with severe tremor had higher creatinine blood concentrations, which is consistent with the notion that tacrolimus is both nephrotoxic and neurotoxic (18, 19). However, alternatively, renal impairment may also predispose patients to tremor, so tacrolimus use does not necessarily relate to the occurrence of both nephro- and neurotoxicity in the same patient and claims regarding causality cannot be drawn from the current study. Nevertheless, our findings support the previously suggested notion that the occurrence of CNI side effects is dose-dependent (18, 19). Generally, factors including vitamin B12, HbA1c and glucose are regarded as key determinants of tremor in different populations (28), and concomitant medication such as bronchodilators and betablockers are known to affect tremor. Our results highlight that these factors appear to be less important among SOTR, and that CNI use appears to be the main cause of tremor in this population.

The notion that tacrolimus trough concentrations are independently associated with tremor-related impairment among SOTR is important for treating physicians to consider during treatment regimens. Furthermore, this notion highlights the need for alternative immunosuppressive treatment regimens that are less likely to cause tremor, while maintaining low risks of rejection. Since dose-dependency seems to be key in the pathophysiology of medication-induced tremor, extended-release preparations may be able to reduce tremor prevalence by reducing peak-to-trough variability (19). Langone et al. have reported promising results in patients using extended-release tacrolimus, showing a significantly lower peak-to-trough variability, lower tremor prevalence, and a higher quality of life (19). However, this study included a small number of only kidney transplant recipients. Therefore, future interventional studies are required for strengthening these results.

The potential impact of tremor on the lives of SOTR is further highlighted by the associations of severe tremor with both lower mental and physical HRQoL. These results are generally in line with findings in previous smaller studies. For example, Langone et al. reported an improved quality of life after a reduction in tremor severity, in a small cohort of 38 kidney transplant recipients (19).

A major strength of this study is the large number of included SOTR with available data regarding subjective tremor and HRQoL. Additionally, we included kidney, liver, lung, heart, and small intestine transplant recipients, allowing for evaluation of tremor prevalence among these patient groups. In addition, the cohort was well-characterized, allowing us to adjust for many potential confounders, including extensive clinical data, laboratory measurements, and treatment regimens. A limitation of the current study is that the participants mainly used tacrolimus-based treatment regimens, and associations of cyclosporine and other immunosuppressive drugs with tremor could therefore be incompletely studied. In addition, since patients were not evaluated before transplantation, the presence and exacerbation of preconditions which may cause tremor could not be assessed. Non-CNI users with tremor more frequently had diabetes compared to those without diabetes, which may partly explain the high prevalence of tremor in this subgroup. Nevertheless, this observation cannot fully explain the tremor occurrence in this subgroup, and future studies are needed to gain insights into the tremor susceptibility of some SOTR without CNI-use. Lastly, due to the observational design of the study, we cannot draw conclusions regarding causality of our findings. Moreover, the current cross-sectional study cannot identify trajectories of tremor before and after transplantation, and therefore longitudinal studies assessing the determinants of tremor are warranted. Such studies may also help to account for potential tacrolimus dosage adaptations that clinicians may conduct in patients with severe tremor.

SOTR frequently report tremor-related impairment of ADL. Tacrolimus trough concentrations appeared a main determinant of tremor among SOTR. The strong and independent association of tremor-related impairment with lower HRQoL warrants further studies into the effects of tacrolimus on tremor.

## Members of Transplantlines Investigators

Coby Annema, Stephan J. L. Bakker, Stefan P. Berger, Hans Blokzijl, Frank A. J. A. Bodewes, Marieke T. de Boer, Kevin Damman, Martin H. de Borst, Arjan Diepstra, Gerard Dijkstra, Caecilia S. E. Doorenbos, Rianne M. Douwes, Michele F. Eisenga, Michele E. Erasmus, C. Tji Gan, Antonio W. Gomes Neto, Eelko Hak, Bouke G. Hepkema, Frank Klont, Tim J. Knobbe, Daan Kremer, Henri G. D. Leuvenink, Willem S. Lexmond, Vincent E. de Meijer, Hubert G. M. Niesters, Gertrude J. Nieuwenhuijs - Moeke, L. Joost van Pelt, Robert A. Pol, Adelita V. Ranchor, Jan Stephan F. Sanders, Marion J. Siebelink, Riemer J. H. J. A. Slart, J. Casper Swarte, Daan J. Touw, Marius C. van den Heuvel, Coretta van Leer-Buter, Marco van Londen, Erik A. M. Verschuuren, Michel J. Vos, Rinse K. Weersma.

## Data Availability

The datasets presented in this article are not readily available because public participant data sharing is not included in the TransplantLines informed consent forms. Data requests are only legally allowed after approval by the TransplantLines Scientific Committee upon reasonable request, in accordance with the medical ethical committee allowance and the UMCG Biobank Regulations. Requests to access the datasets should be directed to the corresponding author.

## Abbreviations

ADL, Activities of daily living; CNI, Calcineurin inhibitor; FTM TRS-C, Fahn-Tolosa-Marin tremor rating scale C; HRQoL, Health-related quality of life; MCS, Mental component summary; PCS, Physical component summary; SOTR, Solid organ transplant recipients.
