# Supplementary material for: Tremor, Daily Functioning, and Health-Related Quality of Life in Solid Organ Transplant Recipients
Source: Transpl Int. 2023 Mar 16;36:10951. doi: 10.3389/ti.2023.10951 (PMC10062599; doi:10.3389/ti.2023.10951)
Supplement: Supplementary file 1 [file DataSheet1.docx]

**Supporting information**

**Supporting Table 1 |** The Fahn-Tolosa-Marín Tremor Rating Scale C that was used for tremor assessment using a Dutch translation

1. **Speaking**

*This includes spastic dysphonia if present*

0 = Normal

1 = Mild voice tremulousness when ‘when nervous’ only

2 = Mild voice tremor, constant

3 = Moderate voice tremor

4 = Severe voice tremor. Some words difficult to understand.

1. **Feeding** (*other than liquids)*

0 = Normal

1 = Mildly normal. Can bring all solids to mouth, spilling only rarely.

2 = Moderately abnormal. Frequent spills of peas and similar foods.

May bring head at least halfway to meet food.

3 = Markedly abnormal. Unable to cut or uses hands to feed.

4 = Severely abnormal. Needs help to feed.

1. **Bringing liquids to mouth**

0 = Normal
1 = Mildly abnormal. Can still use a spoon, but not if it is completely full
2 = Moderately abnormal. Unable to use a spoon; uses cup or glass
3 = Markedly abnormal. Can drink from cup or glass but needs two hands
4 = Severely abnormal. Must use a straw.

1. **Hygiene**

0 = Normal

1 = Mildly abnormal. Able to do everything but is more careful than the average person

2 = Moderately abnormal. Able to do everything, but with errors; uses electric razor

because of tremor

3 = Markedly abnormal. Unable to do most fine tasks, such as putting on lipstick or

Shaving (even with electric razor), unless using two hands.

4 = Severely abnormal. Unable to do any fine-movement tasks.

1. **Dressing**

0 = Normal
1 = Mildly abnormal. Able to do everything but is more careful than the average person.
2 = Moderately abnormal. Able to do everything, but with errors.
3 = Markedly abnormal. Needs some help with buttoning or other activities, such as tying

shoelaces.

4 = Severely abnormal. Requires assistance even for gross motor activities.

1. **Writing**

0 = Normal
1 = Mildly abnormal. Legible. Continues to write letters.
2 = Moderately abnormal. Legible, but no longer writes letters.
3 = Markedly abnormal. Illegible.
4 = Severely abnormal. Unable to sign checks or other documents requiring a signature.

1. **Working**

0 = Tremor does not interfere with job
1 = Able to work but needs to be more careful than the average person
2 = Able to do everything, but with errors. Poorer than usual performance because of tremor
3 = Unable to do regular job. May have changed to a different job because of tremor.

Tremor limits housework, such as ironing.

4 = Unable to do any outside job; housework is very limited.

1. **Social activities**

0 = Normal
1 = Minimal change in social activities, still socializes.
2 = Moderate change in social activities, avoids meeting with strangers.
3 = Marked change in social activities, avoids meeting with friends.
4 = Severe change in social activities, avoids meeting in public.

| **Supporting Table 2 \|** Multinomial logistic regression analyses of tremor with adjustment for age, sex, log_2_ time after transplantation and tacrolimus trough concentrations in 514 SOTR | | | | | | | |
| --- | --- | --- | --- | --- | --- | --- | --- |
|  |  | **No tremor (n=402)** | **Mild tremor (n=206)** | | **Severe tremor (n=81)** | |  |
|  | *Baseline variables* |  | OR (95% CI) | P-value | OR (95% CI) | P-value | N |
| **Recipient** | |  |  |  |  |  |  |
|  | *Female sex | *Ref.* | 0.97 (0.65-1.45) | 0.9 | 1.22 (0.70-2.14) | 0.5 | 514 |
|  | *Age at visit (per 10 years) | *Ref.* | 0.98 (0.85-1.13) | 1.0 | 1.13 (0.92-1.40) | 0.2 | 514 |
|  | *^#^Transplant type* |  |  |  |  |  |  |
|  | Kidney (n=299) | Ref. | Ref. | Ref. | *Ref.* | *Ref.* | - |
|  | Liver (n=108) | Ref. | 1.27 (0.74-2.17) | 0.4 | 0.95 (0.41-2.19) | 0.9 | 514 |
|  | Lung (n=86) | *Ref.* | **1.93 (1.10-3.40)** | **0.024** | 1.90 (0.85-4.25) | 0.1 | 514 |
|  | Heart (n=20) | *Ref.* | 1.32 (0.44-3.40) | 0.6 | **4.21 (1.31-12.95)** | **0.015** | 514 |
|  | Polypharmacy (>4 drugs) | *Ref.* | 1.15 (0.62-2.15) | 0.7 | 2.72 (0.79-9.41) | 0.1 | 514 |
|  | Diabetes | *Ref.* | 0.90 (0.58-1.40) | 0.6 | 0.69 (0.36-1.34) | 0.3 | 514 |
|  | Anaemia | *Ref.* | 1.15 (0.72-1.85) | 0.6 | 1.65 (0.86-3.16) | 0.1 | 425 |
|  | Body mass index (kg/m^2^) | *Ref.* | 0.97 (0.93-1.02) | 0.2 | 0.98 (0.92-1.04) | 0.4 | 512 |
| **Kidney transplant characteristics** | |  |  |  |  |  |  |
|  | Log_2_ donor age (years) | *Ref.* | 1.10 (0.77-1.58) | 0.6 | 0.78 (0.49-1.23) | 0.3 | 483 |
|  | Living donor | *Ref.* | 0.85 (0.57-1.26) | 0.4 | 0.58 (0.31-1.09) | 0.6 | 514 |
|  | Log_2_ time after transplantation (years) | *Ref.* | 1.00 (0.86-1.15) | 1.0 | 1.01 (0.82-1.25) | 0.9 | 514 |
| **Laboratory measurements** | |  |  |  |  |  |  |
|  | eGFR creatinine (mL/min/1.73m2) | *Ref.* | 1.00 (0.97-1.01) | 0.4 | 0.99 (0.97-1.01) | 0.2 | 478 |
|  | Log_2_ creatinine (µmol/L) | *Ref.* | 1.31 (0.85-2.01) | 0.2 | **2.01 (1.15-3.53)** | **0.015** | 478 |
|  | Haemoglobin (mmol/L) | *Ref.* | 0.93 (0.77-1.13) | 0.5 | 0.86 (0.65-1.13) | 0.3 | 477 |
|  | Log_2_ HbA1c (mmol/mol) | *Ref.* | 1.48 (0.78-2.81) | 0.2 | 1.15 (0.44-3.01) | 0.8 | 476 |
|  | Log_2_ glucose (mmol/L) | *Ref.* | 1.59 (0.86-2.95) | 0.1 | 1.88 (0.81-4.41) | 0.1 | 462 |
|  | Log_2_ vitamin B12 (pmol/L) | *Ref.* | 1.07 (0.80-1.43) | 0.7 | **1.50 (1.01-2.23)** | **0.047** | 469 |
|  | Log_2_ folic acid (nmol/L) | *Ref.* | 0.93 (0.69-1.24) | 0.6 | 1.18 (0.77-1.80) | 0.4 | 435 |
|  | *Tacrolimus (µg/L) | *Ref.* | **1.11 (1.02-1.21)** | **0.011** | 1.11 (0.97-1.24) | 0.1 | 514 |
| **Medication** | |  |  |  |  |  |  |
|  | *~Calcineurin inhibitor use* | *Ref.* | - | - | - | - | - |
|  | mTOR inhibitor | *Ref.* | 1.12 (0.43-2.92) | 0.8 | 2.60 (0.85-7.93) | 0.1 | 514 |
|  | Proliferation inhibitor use | *Ref.* | 0.94 (0.57-1.53) | 0.8 | 1.19 (0.57-2.50) | 0.6 | 514 |
|  | Prednisolone or prednisone | *Ref.* | 1.04 (0.62-1.75) | 0.9 | 1.65 (0.71-3.83) | 0.2 | 514 |
|  | Beta blockers | *Ref.* | 0.88 (0.59-1.30) | 0.5 | 1.05 (0.59-1.85) | 0.9 | 513 |
|  | Short or long acting bronchodilators | *Ref.* | 0.80 (0.31-2.05) | 0.6 | 1.61 (0.55-4.72) | 0.4 | 514 |
| Bolt type indicates significance of results. Log_2_, the binary logarithm; eGFR, estimated glomerular filtration rate as calculated using CKD-EPI formula. *Only adjusted for the other variables. ^#^Intestinal transplant recipients are excluded in analyses due to low number of participants (n=2). ^~^Cyclosporine concentrations and calcineurin inhibitor use were excluded in analyses because only patients with data on tacrolimus trough concentration were included in the model. | | | | | | | |

| **Supporting Table 3** \| Characteristics of 95 non-CNI users without tremor, with mild tremor or with severe tremor, based on the TRS-C total score | | | | | | | | | |
| --- | --- | --- | --- | --- | --- | --- | --- | --- | --- |
| *Variables* | | **No tremor (n=61)** | **Mild tremor (n=23)** | | **Severe tremor (n=11)** | | P-value | |  |
| Tremor rating scale-C score | | 0.0 [0.0-0.0] | 2.0 [1.0-2.0] | | 4.0 [4.0-5.0] | | - | |  |
| **Recipient** | |  | |  | |  | |  | |
|  | Female sex | 22 (36.1%) | 8 (34.8%) | | 6 (54.5%) | | 0.5 | |  |
|  | Age at visit (years) | 54.9 ± 13.9 | 60.5 ± 12.3 | | 57.3 ± 17.7 | | 0.3 | |  |
|  | *Transplant type^#^* |  |  | |  | |  | |  |
|  | Kidney (n=47) | 26 (42.7%) | 13 (56.5%) | | 8 (72.7%) | | 0.1 | |  |
|  | Liver (n=45) | **34 (55.7%)** | **9 (39.1%)** | | **2 (18.2%)** | | **0.047** | |  |
|  | Lung (n=2) | 1 (1.6%) | 1 (4.3%) | | 0 (0.0%) | | 0.7 | |  |
|  | Heart (n=0) | 0 (0.0%) | 0 (0.0%) | | 0 (0.0%) | | - | |  |
|  | Small intestine (n=1) | **0 (0.0%)** | **0 (0.0%)** | | **1 (9.1%)** | | **0.021** | |  |
|  | Polypharmacy (>4 drugs) | 45 (73.8%) | 19 (82.6%) | | 10 (90.9%) | | 0.4 | |  |
|  | Diabetes | **10 (16.4%)** | **7 (30.4%)** | | **6 (54.5%)** | | **0.018** | |  |
|  | Anaemia | **4 (8.7%)** | **7 (35.0%)** | | **2 (18.2%)** | | **0.032** | |  |
|  | Body mass index (kg/m^2^) | 26.1 ± 5.1 | 27.7 ± 4.6 | | 26.7 ± 5.5 | | 0.4 | |  |
| **Kidney transplant characteristics** | |  | |  | |  | |  | |
|  | Donor age (years) | 43.0 [32.8-54.3] | 45.0 [33.0-61.0] | | 45.0 [27.5-52.0] | | 0.7 | |  |
|  | Living donor | 13 (21.3%) | 7 (30.4%) | | 5 (45.5%) | | 0.2 | |  |
|  | Time after transplantation (years) | 13.0 [7.5-21.5] | 13.0 [9.0-21.0] | | 19.0 [12.0-22.0] | | 0.3 | |  |
| **Laboratory measurements** | |  | |  | |  | |  | |
|  | eGFR creatinine (mL/min/1.73m2) | **68.3 ± 25.1** | **56.2 ± 16.1** | | **73.3 ± 15.3** | | **0.048** | |  |
|  | Creatinine (µmol/L) | 95.0 [76.0-125.0] | 116.0 [102.0-126.0] | | 86.0 [72.0-115.0] | | 0.1 | |  |
|  | Haemoglobin (mmol/L) | 8.7 ± 1.1 | 8.3 ± 1.1 | | 8.4 ± 0.9 | | 0.3 | |  |
|  | HbA1c (mmol/mol) | 36.0 [32.0-40.0] | 40.0 [37.0-44.0] | | 40.0 [34.0-65.0] | |  | |  |
|  | Glucose (mmol/L) | 5.4 [5.0-5.9] | 5.5 [4.8-6.6] | | 6.4 [5.0-11.6] | | 0.4 | |  |
|  | Vitamin B12 (pmol/L) | 323.5 [248.3-395.5] | 324.0 [253.0-431.0] | | 222.0 [209.0-347.0] | | 0.2 | |  |
|  | Folic acid (nmol/L) | 13.4 [9.8-18.1] | 17.0 [11.9-24.7] | | 12.9 [10.1-22.4] | | 0.2 | |  |
| **Medication** | |  | |  | |  | |  | |
|  | mTOR inhibitor | 6 (9.8%) | 6 (26.1%) | | 0 (0.0%) | | 0.1 | |  |
|  | Proliferation inhibitor | 53 (86.9%) | 22 (95.7%) | | 11 (100.0%) | | 0.2 | |  |
|  | Prednisolone or prednisone | 54 (88.5%) | 21 (91.3%) | | 11 (100.0%) | | 0.5 | |  |
|  | Beta blockers | 23 (37.7%) | 11 (47.8%) | | 3 (27.3%) | | 0.5 | |  |
|  | Short or long acting bronchodilators | 3 (4.9%) | 3 (13.0%) | | 0 (0.0%) | | 0.3 | |  |
| Bolt type indicates significance of results. eGFR, estimated glomerular filtration rate as calculated using CKD-EPI formula. Normally distributed data are presented as mean ± standard deviation, skewed data as median [interquartile range], and categorical data as number (valid percentage). P-values represent significance of differences between tremor severity groups as assessed with Analyses of Variance, Kruskal-Wallis or Chi-squared tests, depending on distribution. ^#^Percentages were calculated by dividing the number of patients in each transplant type by the total number of all solid organ transplant patients with no/mild/severe tremor. | | | | | | | | | |

**
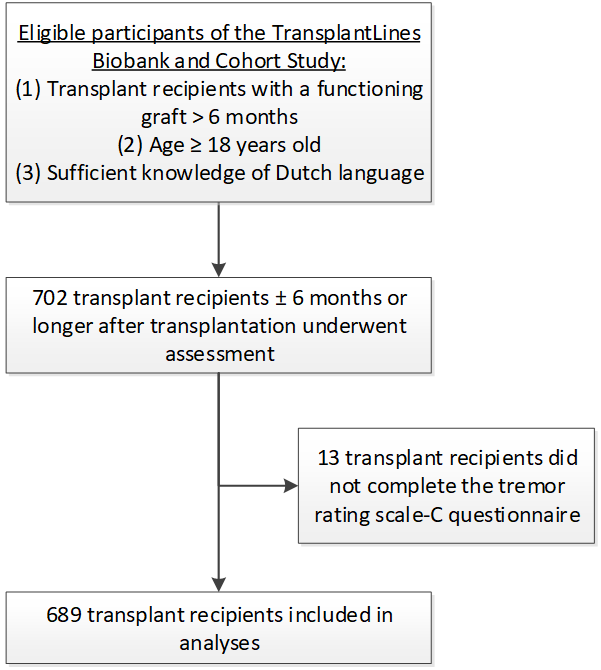
**

**Supporting Figure 1** | Consort flow diagram


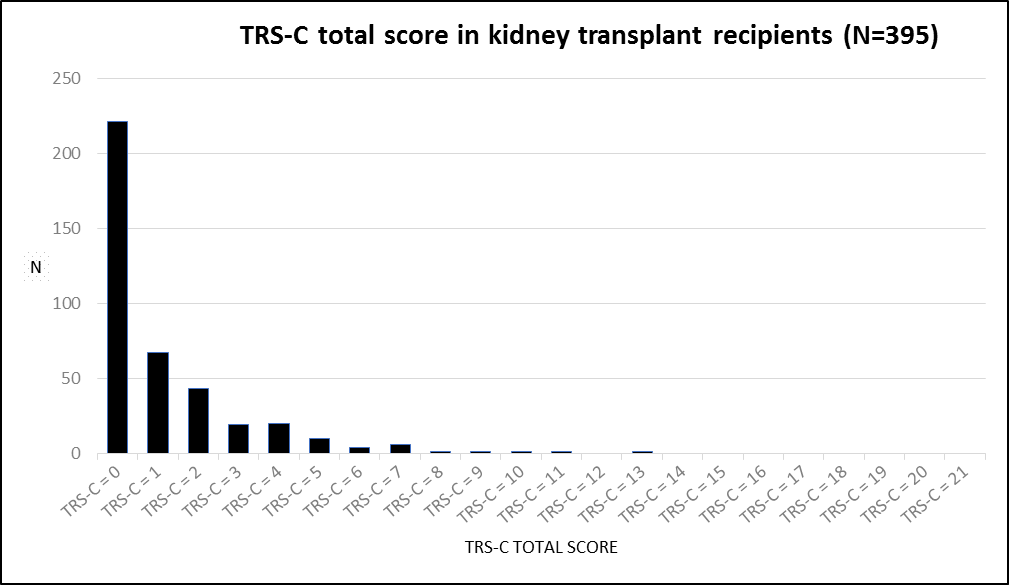


**Supporting Figure 2A** | Bar chart of TRS-C total score in 395 kidney transplant recipients. Abbreviations: TRS-C: tremor rating scale part C; SOTR: solid organ transplant recipients.


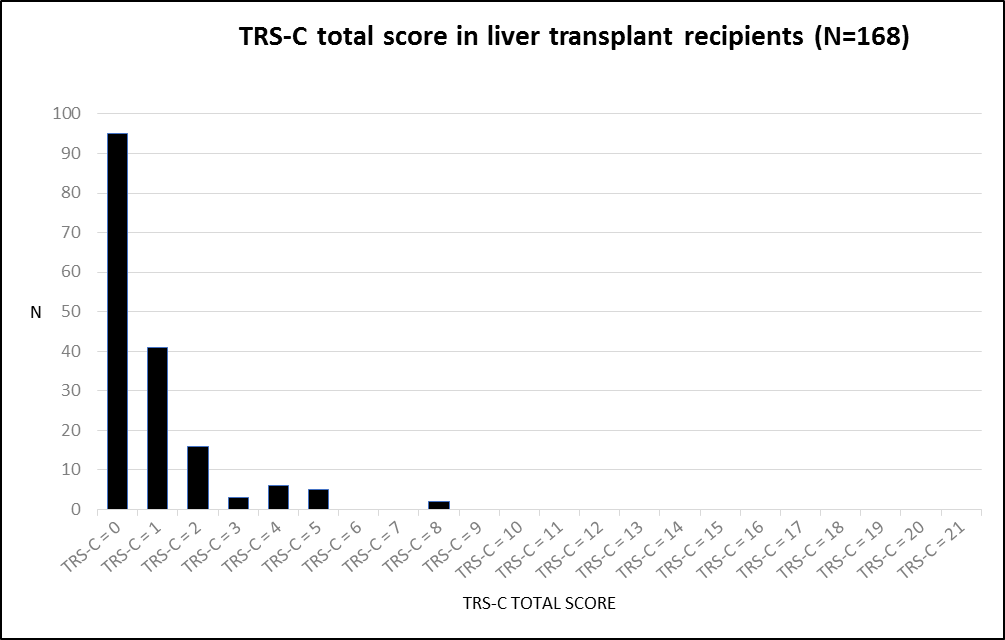


**Supporting Figure 2B** | Bar chart of TRS-C total score in 168 liver transplant recipients. Abbreviations: TRS-C: tremor rating scale part C.


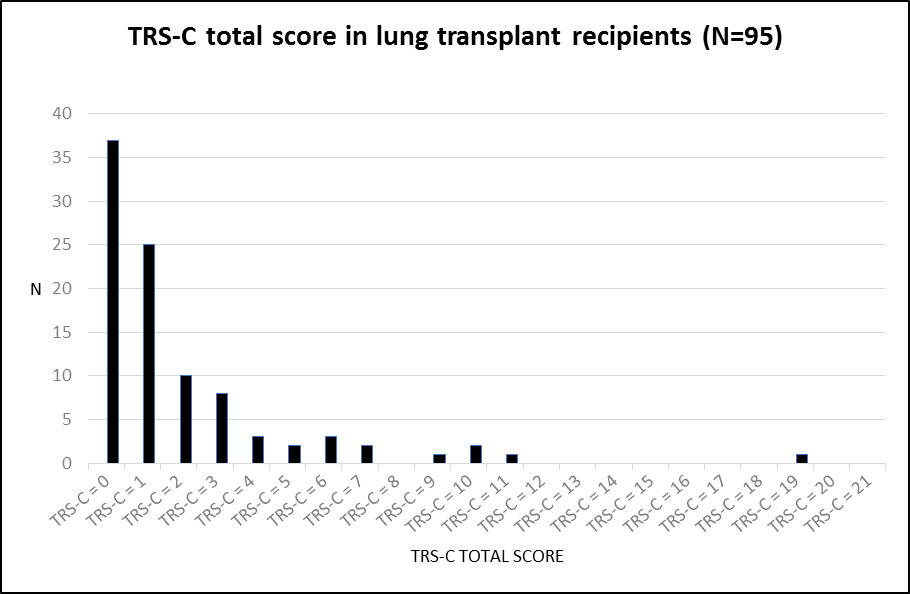


**Supporting figure 2C** | Bar chart of TRS-C total score in 95 lung transplant recipients. Abbreviations: TRS-C: tremor rating scale part C.


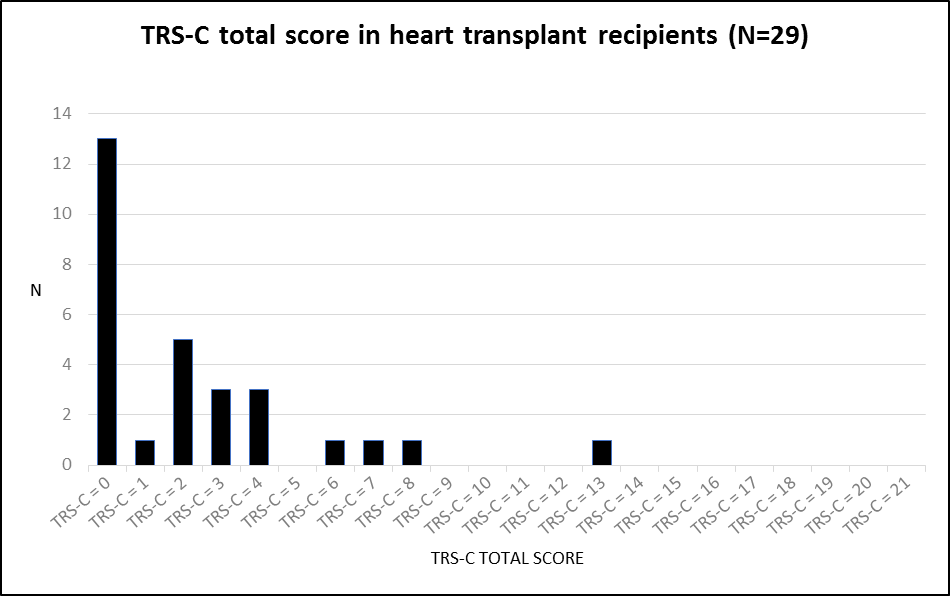


**Supporting Figure 2D** | Bar chart of TRS-C total score in 29 heart transplant recipients. Abbreviations: TRS-C: tremor rating scale part C.

**
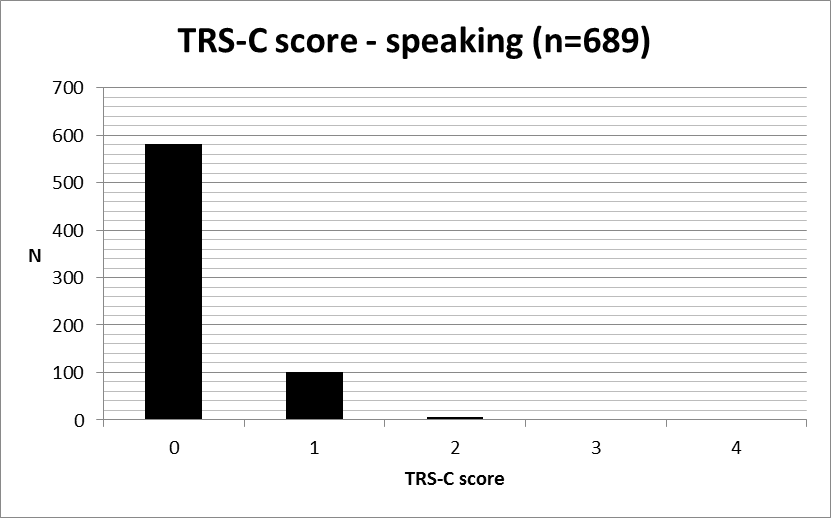
**

**Supporting Figure 3A** | Bar chart of TRS-C score in 689 solid organ transplant recipients – speaking. Abbreviations: TRS-C: tremor rating scale part C.


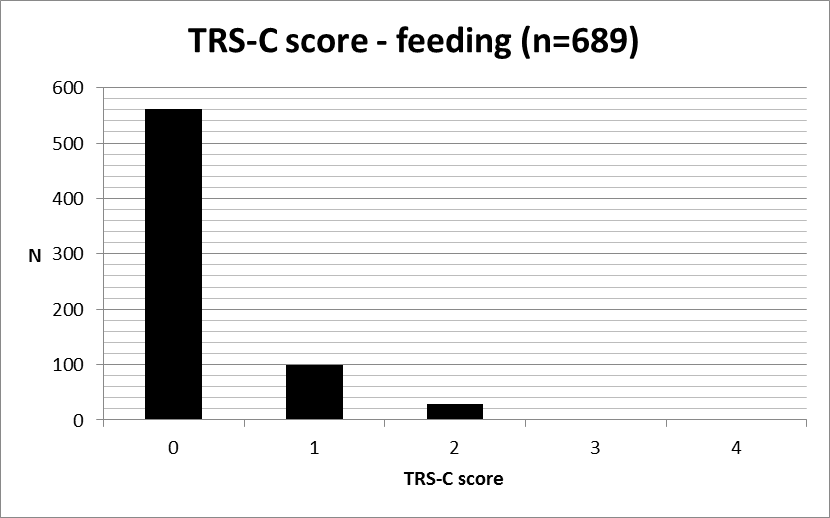


**Supporting figure 3B** | Bar chart of TRS-C score in 689 solid organ transplant recipients – feeding. Abbreviations: TRS-C: tremor rating scale part C.


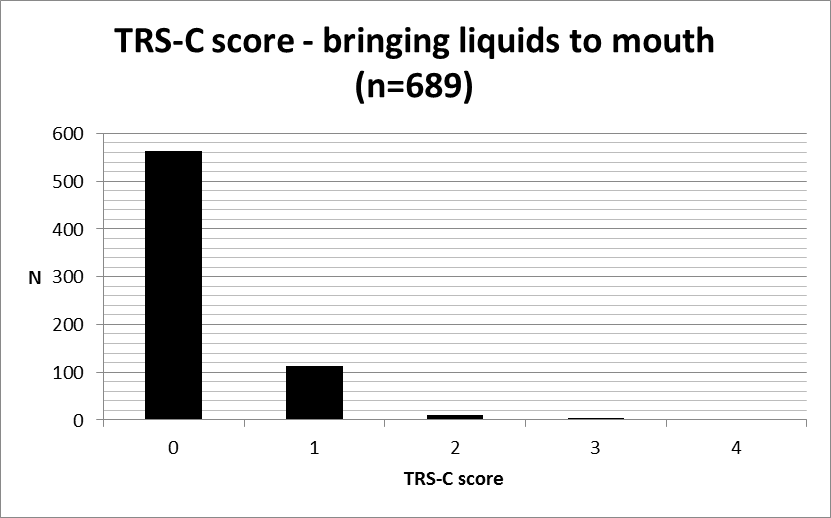


**Supporting Figure 3C** | Bar chart of TRS-C score in 689 solid organ transplant recipients – bringing liquids to mouth. Abbreviations: TRS-C: tremor rating scale part C.


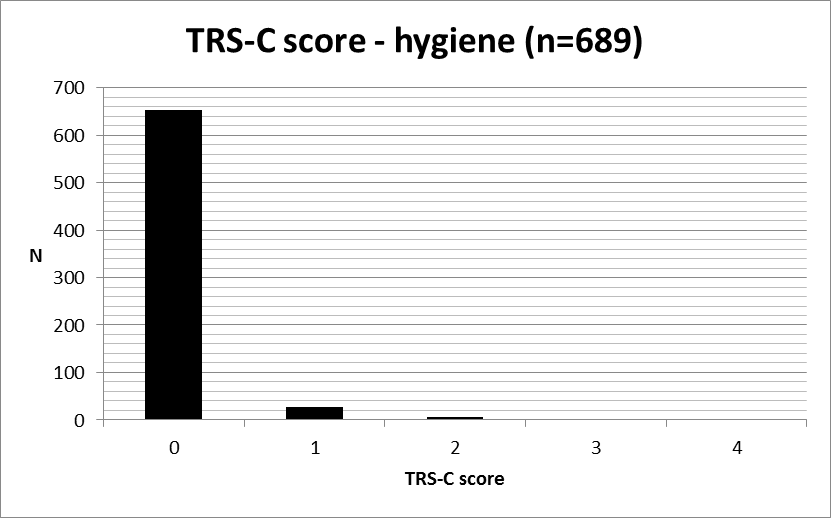


**Supporting Figure 3D** | Bar chart of TRS-C score in 689 solid organ transplant recipients – hygiene. Abbreviations: TRS-C: tremor rating scale part C.


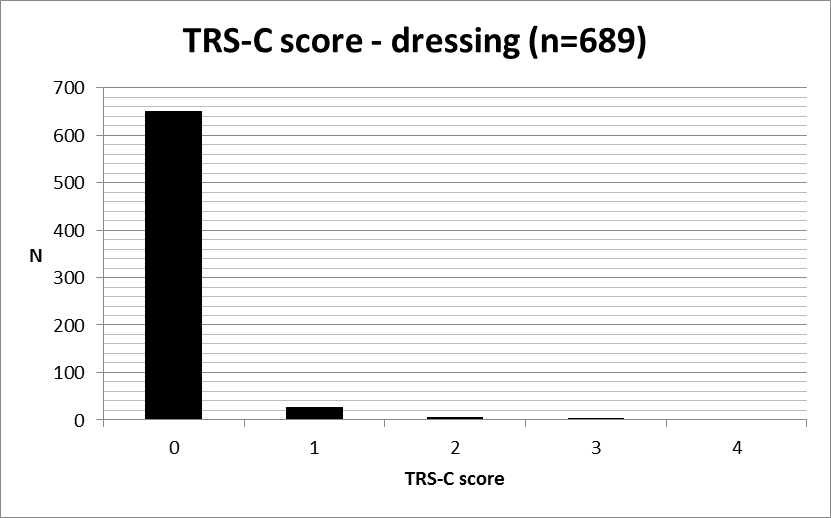


**Supporting Figure 3E** | Bar chart of TRS-C score in 689 solid organ transplant recipients – dressing. Abbreviations: TRS-C: tremor rating scale part C.


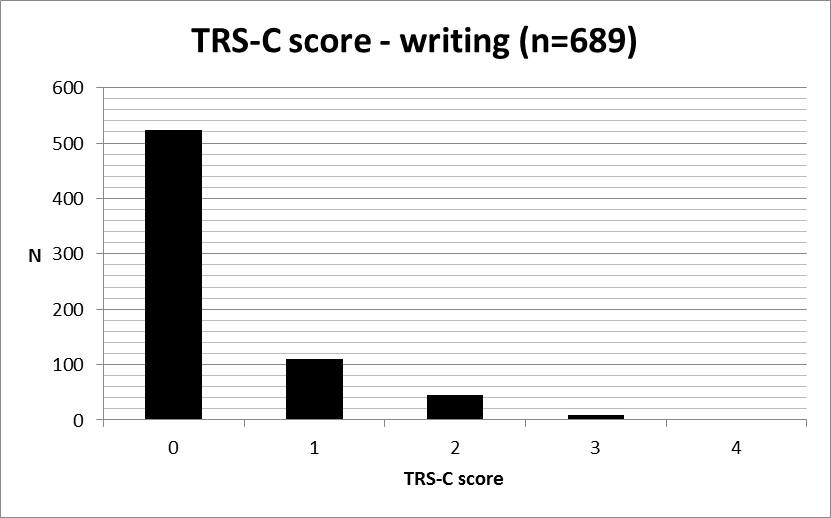


**Supporting Figure 3F** | Bar chart of TRS-C score in 689 solid organ transplant recipients – writing. Abbreviations: TRS-C: tremor rating scale part C.


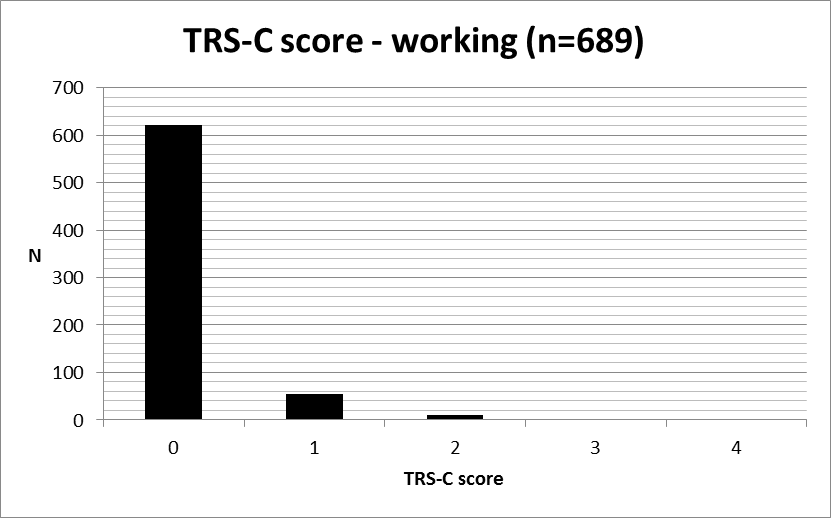


**Supporting Figure 3G** | Bar chart of TRS-C score in 689 solid organ transplant recipients – working. Abbreviations: TRS-C: tremor rating scale part C.


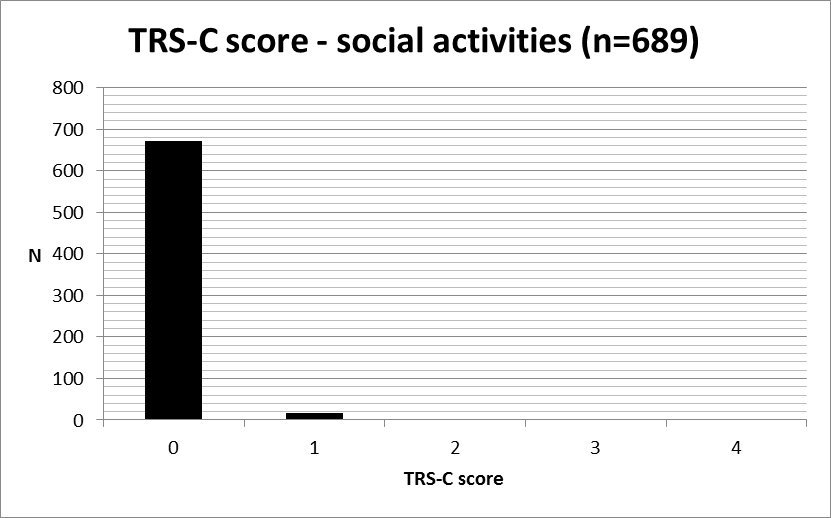


**Supporting Figure 3H**| Bar chart of TRS-C score in 689 solid organ transplant recipients – social activities. Abbreviations: TRS-C: tremor rating scale part C.
